# Metabolic changes during respiratory syncytial virus infection of epithelial cells

**DOI:** 10.1371/journal.pone.0230844

**Published:** 2020-03-26

**Authors:** María Martín-Vicente, Carolina González-Riaño, Coral Barbas, María Ángeles Jiménez-Sousa, Oscar Brochado-Kith, Salvador Resino, Isidoro Martínez

**Affiliations:** 1 Unidad de Infección Viral e Inmunidad, Centro Nacional de Microbiología, Instituto de Salud Carlos III, Madrid, Spain; 2 Facultad de Farmacia, Centro de Metabolómica y Bioanálisis (CEMBIO), Universidad CEU San Pablo, Madrid, Spain; Oswaldo Cruz Foundation, BRAZIL

## Abstract

Viral infections induce substantial metabolic changes in infected cells to optimize viral production while cells develop countermeasures to restrict that infection. Human respiratory syncytial virus (HRSV) is an infectious pathogen that causes severe lower respiratory tract infections (LRTI) in infants, the elderly, and immunocompromised adults for which no effective treatment or vaccine is currently available. In this study, variations in metabolite levels at different time points post-HRSV infection of epithelial cells were studied by untargeted metabolomics using liquid chromatography/mass spectrometry analysis of methanol cell extracts. Numerous metabolites were significantly upregulated after 18 hours post-infection, including nucleotides, amino acids, amino and nucleotide sugars, and metabolites of the central carbon pathway. In contrast, most lipid classes were downregulated. Additionally, increased levels of oxidized glutathione and polyamines were associated with oxidative stress in infected cells. These results show how HRSV infection influences cell metabolism to produce the energy and building blocks necessary for virus reproduction, suggesting potential therapeutic interventions against this virus.

## Introduction

The relevance of metabolomics studies is increasingly recognized in many research areas, including host-pathogen interactions. Accordingly, the impact of viral infection on host metabolism has been analyzed for several DNA and RNA viruses [1]. Viruses are intracellular parasites that rely on cellular metabolism to obtain all the necessary structural and energetic resources for its replication. Nucleotides, amino acids, lipids, and sugars are expropriated from the intracellular pool and incorporated into the new virions. The energy necessary for this process is also provided by the cell. Thus, it is not surprising that viruses and infected cells have co-evolved to develop measures and countermeasures to promote or restrict virus replication, respectively. This battle results in a profound impact of virus infection on the host cell’s metabolism [1–3]. Also, this dependence of viruses on cell metabolism provides new intervention opportunities for limiting virus replication.

The human respiratory syncytial virus (HRSV) is an enveloped, negative-sense single-stranded RNA virus that belongs to the *Pneumoviridae* family [4]. HRSV infects people of all ages, but severe pathologies (bronchiolitis and pneumonia) are observed mostly in infants, the elderly, and immunocompromised adults [5, 6]. Recent studies have reported that, globally, HRSV causes more than 33 million lower respiratory tract infections (LRTI) per year in children under five, which results in about 3 million hospitalizations and around 60,000 deaths [7, 8]. HRSV infection is also related to the development of asthma and the exacerbation of chronic obstructive pulmonary disease (COPD) [9, 10]. At present, there is no licensed HRSV vaccine available or effective treatment against HRSV. A humanized neutralizing monoclonal antibody (Palivizumab, Synagis) directed to the fusion protein F of the virus is used in high-risk individuals, but it is only effective when administered prophylactically [11].

Transcriptomic and proteomic studies have shown the profound impact of HRSV on the physiology of respiratory epithelial cells, its main targets for infection. However, most of those studies have focused on the early innate immune response elicited in the infected cells [12–14]. Although, changes in other aspects of the virus-host cell interactions have also been reported, including those involving cell growth, cytoskeleton organization and mitochondrial electron transport [15–18]. Metabolic studies may complement transcriptomic an proteomic analysis unraveling new aspects of virus-host interactions and, therefore, new potential intervention strategies. Metabolomic analyses on HRSV infection have been conducted only very recently, mostly in the biological fluids of infected patients [19–24]. An alternative to analyzing biofluids from infected individuals is to use cellular models in more controlled experimental settings, such as cultured cells that directly support the entire viral replication cycle.

In this study, our objective was to evaluate the impact of HRSV infection on the physiology of infected epithelial cells by analyzing the time-dependent metabolic changes induced by the virus on those cells. Our results show how HRSV manipulates cell metabolism to obtain the energy and structural components to ensure virus production, including the upregulation of glycolysis and citric acid cycle components, nucleotides, amino acids, and amino and nucleotide sugars. In contrast, most lipids were downregulated in infected cells. Similarly as to what has been reported in previous studies, HRSV infection also led to increased oxidative stress [25–29].

## Material and methods

### Cells and virus

Epithelial cells and HRSV were cultured as previously described [30]. In brief, Human lung carcinoma cells (A549, ATCC® CCL-185) and human carcinoma HeLa-derived cells (HEp-2, ATCC® CCL-23) were grown in Dulbecco’s modified Eagle’s medium (DMEM, Hyclone, Logan, Utah, USA) complemented with 10% fetal bovine serum (FBS, Biological Industries, Beit HaEmerk, Israel), 4 mM L-Glutamine (HyClone), 100 U/ml penicillin (Lonza, Verviers, Belgium) and 100 μg/ml streptomycin (HyClone) (DMEM10). Cells were maintained at 37°C in a humidified atmosphere containing 5% CO_2_.

Viral stocks of the HRSV Long strain (ATCC® VR-26) were obtained from clarified culture supernatants from HEp-2 infected cells by polyethylene glycol precipitation and centrifugation in a discontinuous sucrose gradient as previously described [15, 31].

### Viral infections and sample preparation

A549 subconfluent monolayers were infected with HRSV at a multiplicity of infection (MOI) of 3 plaque-forming units (pfu) per cell in DMEM with 2% FBS (DMEM2) and incubated for 90 minutes at 37°C, after which additional culture medium was added. Samples were collected at different times post-infection as follows: culture supernatants were removed, cell monolayers were washed two times with PBS and scraped off in cold methanol. The cells were subjected to two freeze-thaw cycles in liquid nitrogen, centrifuged at 6000 x g at 4°C for five minutes, and supernatants were stored at -80°C. As a control, mock-infected cells were included in each experiment and processed in the same way as the infected cells. Time 0 hpi was collected after 90 minutes of viral adsorption.

Processed samples were shipped to the Plataforma de Metabolómica, Centro de Edafología y Biología del Segura-Consejo Superior de Investigaciones Científicas, Murcia, Spain, for futher processing.

Culture extract samples (800uL) were thawed at 4°C and then were centrifuged at 5725 x g for five minutes. The supernatant was filtered through 0.2 μm Millex-HV filter units (Millipore, Billerica, MA) and transferred to amber glass vials containing inserts for UPLC-qTOF-MS analysis. Two quality controls (QC) were used: MS grade water samples and Triclosan-d3 + Scopolamine solution (0.1 μg/mL), which were injected seven times during the batch (beginning, middle and end both in positive and negative mode).

### Nontargeted metabolomics fingerprinting

Equipment and software and UPLC-HR-TOF-MS conditions have been described previously by Telving et al. (2016) [32] and are presented here with minor modifications.

#### Equipment and software

Chromatography was carried out employing an ACQUITY I-Class UPLC system (Waters Corporation, Milford, MA, USA). The mass spectrometer was a Bruker maXis Impact QTOF (Bruker Daltonics, Bremen, Germany). The software employed for instrument control and to acquire HR-TOF-MS data was HyStar 3.2 (Bruker Daltonics) and OTOFcontrol 3.4 (Bruker Daltonics).

For mass calibration, handling of data, and generating a text file (CSV) including the positive findings, the software DataAnalysis 4.2 was used. The external calibrant solution was supplied by a KNAUER Smartline Pump 100 with a pressure sensor (KNAUER, Berlin, Germany).

#### UPLC-HR-TOF-MS analytical conditions

Analytical conditions were published previously by Telving et al. [32], and were applied here with minor modifications. UPLC separation was performed at 30°C on an analytical column from Waters (ACQUITY UPLC HSS T3, 1.8 mm, 2.1 mm x 100 mm) employing gradient elution with mobile phases A (H_2_O + 0.1% formic acid) and B (MeOH + 0.1% formic acid).

The gradient was: initial 0% B, linear to 1% B at 1.0 min, linear to 99% B at 17.0 min, constant 99% B to 19 min, linear to 99% B at 19.5 min, constant 99% B to 20.0 min, immediately down to 1% B, and constant 0% B to 23 min. The flow rate was 0.3 mL/min, and 3 μL of the sample was injected using a flow-through needle (FTN) injection with a 15 μL needle. The sample compartment in the autosampler was maintained at 6.0°C.

Mass spectrometry was carried out using HR-TOF-MS in positive and negative electrospray ionization mode by broadband collision-induced dissociation (bbCID). The m/z range was 50–1200. Nitrogen was employed for the nebulizer, drying gas, and collision gas. High and low collision energy data were collected at the same time by alternating the acquisition between MS “full scan” and bbCID conditions. The instrument was calibrated externally before each sequence with a 10 mM sodium formate solution. The mixture was made by adding 0.1 mL formic acid and 0.5 mL sodium hydroxide to an isopropanol/Milli-Q water solution (1:1, v/v).

### Data processing and multivariate statistical analysis

Each LC-MS data set was processed using the Find Molecular Features (FMF) algorithm in the ProfileAnalysis 2.1 software (Bruker Daltonik, Bremen, Germany) to create a feature list for statistical analysis. Each feature in an LC-MS data set is described by its retention time (RT), m/z value, and its intensity. The parameters of the FMF algorithm were set to the following values: S/N (signal to noise) threshold: 5; correlation coefficient threshold: 0.7; minimum compound length: 10; and smoothing width: 1; MS spectra type: line spectra.

To process the batches of LC-MS data, a transformation into a tabular format, called bucketing, was required. In our analyses, the retention time range was (0.35–17.5 min), and the mass range was (50–1200 Da). The bucket was placed according to its RT and m/z.

The bucket intensity values were normalized (based on the number of cells) to the largest bucket value in each sample. The normalization step is important to ensure comparative parameters across different samples. Retention time alignment was performed with an algorithm from Podwojski and colleagues taking non-linear retention time shifts into account [33].

The data matrices obtained after both LC-MS analyses were first filtered by the metabolic features present in at least 80% of samples per group. Then, the missing values were replaced by k-means nearest neighbor imputation (kNN) [34]. Differences between profiles of infected and control groups were evaluated with univariate data analysis. First, the Shapiro-Wilk test was applied for normality testing, and results showed that the data did not follow a normal distribution. The differences between groups were then evaluated for each metabolite by performing the non-parametrical Mann–Whitney U test (p ≤ 0.05) using MATLAB (R2015a, MathWorks) to conclude whether the metabolite was significant or not in comparison. Finally, the Benjamini–Hochberg correction test was applied to control the false positive rate at level α = 0.05.

Tentative annotation of the metabolites presenting statistically significant differences was achieved by matching the accurate m/z with different databases available online. The tool employed was the search engine CEU Mass Mediator (CMM) [35, 36] that comprises 332,665 real compounds integrated from several metabolomics databases, including HMDB [37], METLIN [38], KEGG [39], and LIPID MAPS [40]. The annotation was based on (i) mass accuracy (maximum error mass was set on 20 ppm); (ii) retention time; (iii) possibility of cation and anion formation; and (iv) adduct formation [41].

Unsupervised principal component analysis (PCA) and enrichment analyses for identified metabolites were performed with MetaboAnalyst 4.0 (www.metaboanalyst.ca) [42]. The heatmaps showing the levels of expression of the identified metabolites were created with Heatmapper (www.heatmapper.ca) (clustering method: “average linkage”, and distance measurement method: “Pearson”) [43].

## Results and discussion

### Time-dependent effects of HRSV on cell metabolism

**Fig 1:**
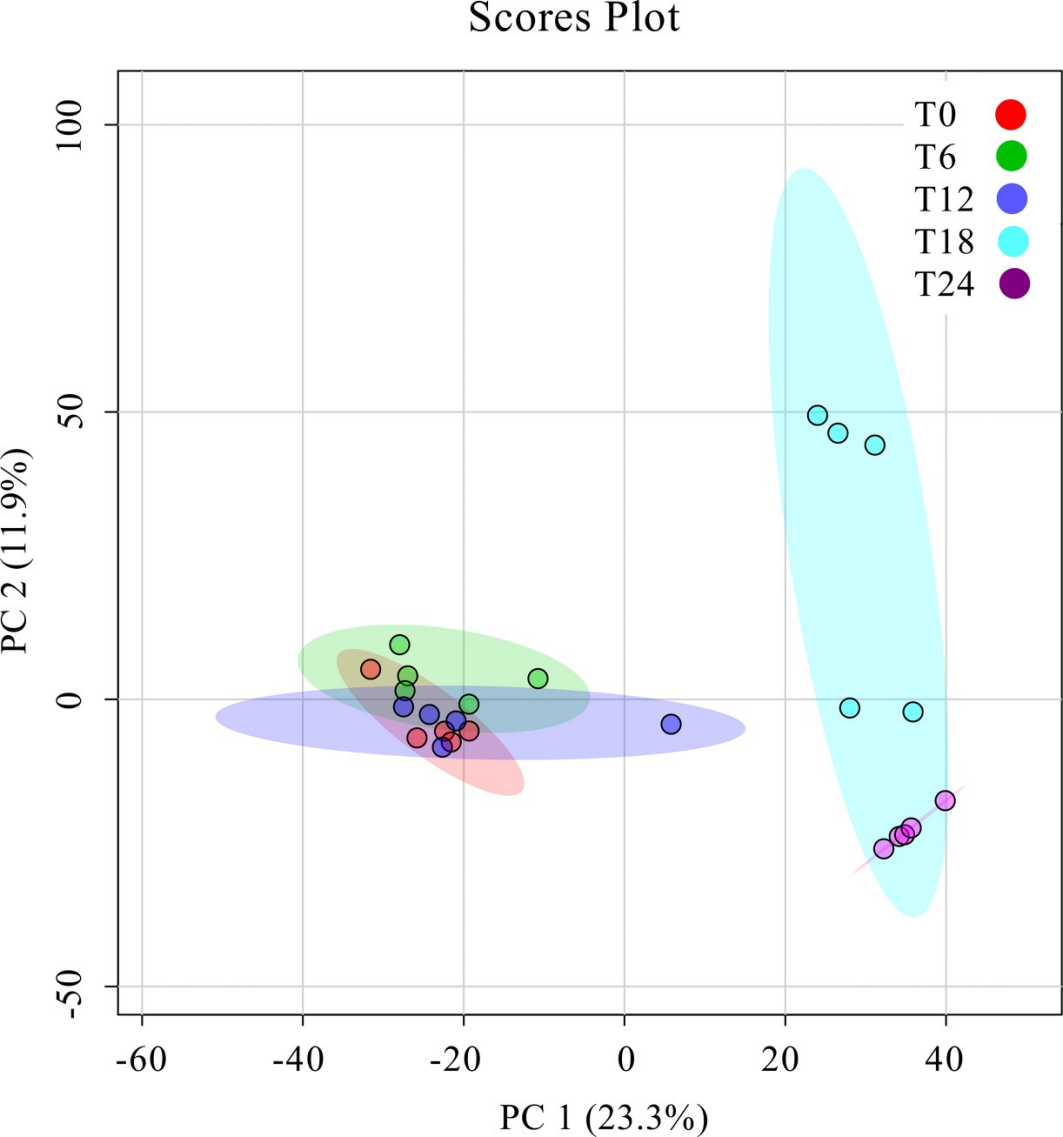
Principal component analysis (PCA) plot. The clustering of samples obtained at different times post-infection is shown. Five independent experiments were carried out for each time point. T0, T6, T12, T18, and T24 (0, 6, 12, 18 and 24 hpi). PC: principal component.

**Fig 2:**
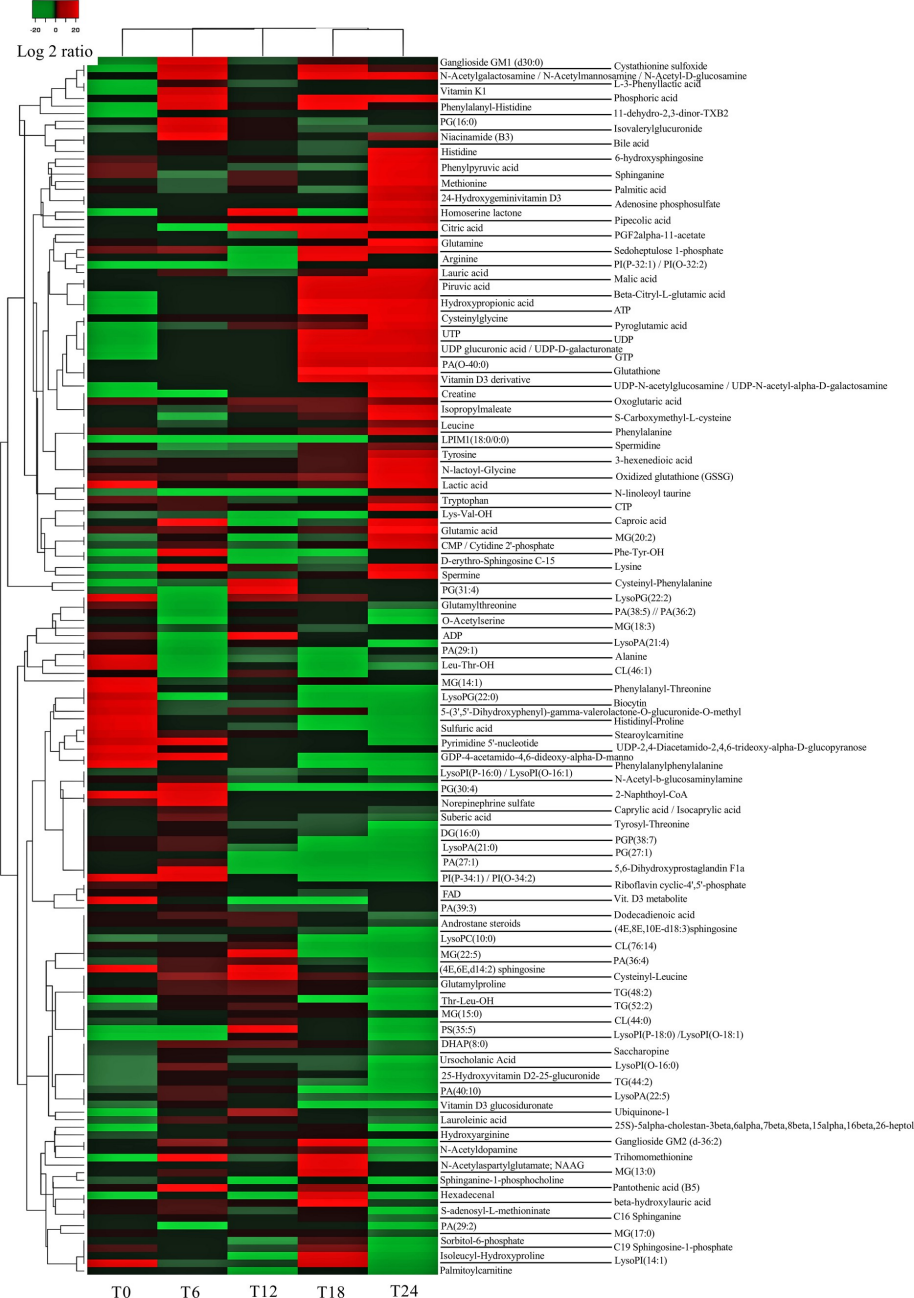
Levels of metabolites in cellular extracts. Metabolites presenting statistically significant differences between infected and mock-infected cells were selected, and a heatmap was created with the identified metabolites. The heatmap shows the median levels from five independent experiments for each time point (0, 6, 12, 18 and 24 hpi). Individual values from the five independent experiments are represented in **S1 Fig**. Levels correspond to the compound area and are represented as log_2_(infected/uninfected).

Previous results from our group showed that HRSV infection had a profound time-dependent impact on the gene expression of infected cells, including the upregulation of many genes involved in protein biosynthesis, amino acid metabolism, response to oxidative stress, vitamin biosynthesis, RNA metabolism, cellular lipid metabolism, etc. [15]. In the present study, we have focused on the metabolic changes induced by HRSV from 0 to 24 hours post-infection (hpi) of epithelial cells. A time-dependent pattern was also observed in this case, and principal component analysis (PCA) separated the different times of infection into two main groups, the early-medium (0, 6 and 12 hpi) and the late (18 and 24 hpi) times (**Fig 1**), which was also evident in a heatmap representation of metabolite levels in infected cells (**Fig 2, S1 Fig**). Most apparent changes took place after 18 hpi (**Fig 2, S1 Fig**), and involved several aspects of the cellular metabolism, as described below. Fold-enrichment analyses were statistically significant (FDR ≤ 0.1) only for 18 and 24 hpi and for the metabolites that increased in infected cells (**S1A and S1B File)**.

### Central carbon metabolism

**Fig 3:**
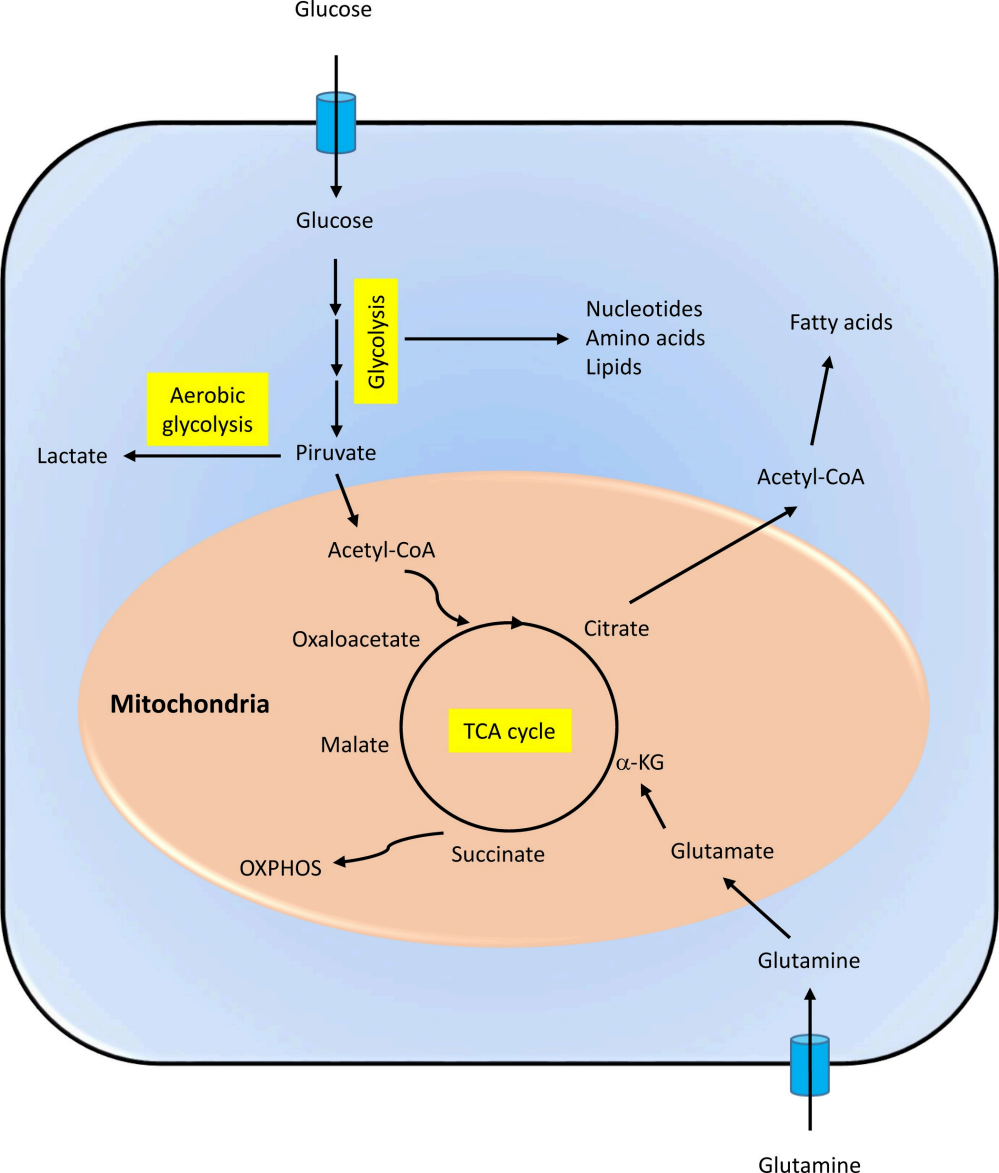
Schematic representation of the central metabolic pathways discussed in the study. α-KG: alpha-ketoglutarate; OXPHOS: oxidative phosphorylation; Acetyl-CoA: acetyl-coenzyme A; TCA: tricarboxylic acid.

Infected cells need to produce high amounts of energy to support active virus replication. Therefore, viruses have evolved to potentiate cellular metabolic pathways from which that energy is generated [1–3]. Most cell energy is produced through the central carbon metabolism pathways, which include glycolysis and the tricarboxylic acid cycle (TCA cycle or citric acid cycle) that drives the electron transport chain to generate ATP in a process termed oxidative phosphorylation (**Fig 3**). These pathways generate the energy necessary for the cellular processes through oxidation of glucose, glutamine, and fatty acids. The primary carbon sources in cell culture are glucose and glutamine, and many viruses increase cellular uptake of both during infection, including human cytomegalovirus (HCMV), Epstein-Barr virus (EBV), Kaposi’s sarcoma-associated herpesvirus (KSHV), human immunodeficiency virus (HIV), rhinovirus, and influenza virus [2, 44]. However, vaccinia virus is an exception, since it requires glutamine, but not glucose, for replication in cell culture [45]. In addition, glycolysis and the TCA cycle produce most of the precursors for the synthesis of amino acids, nucleotides, and lipids.

**Fig 4:**
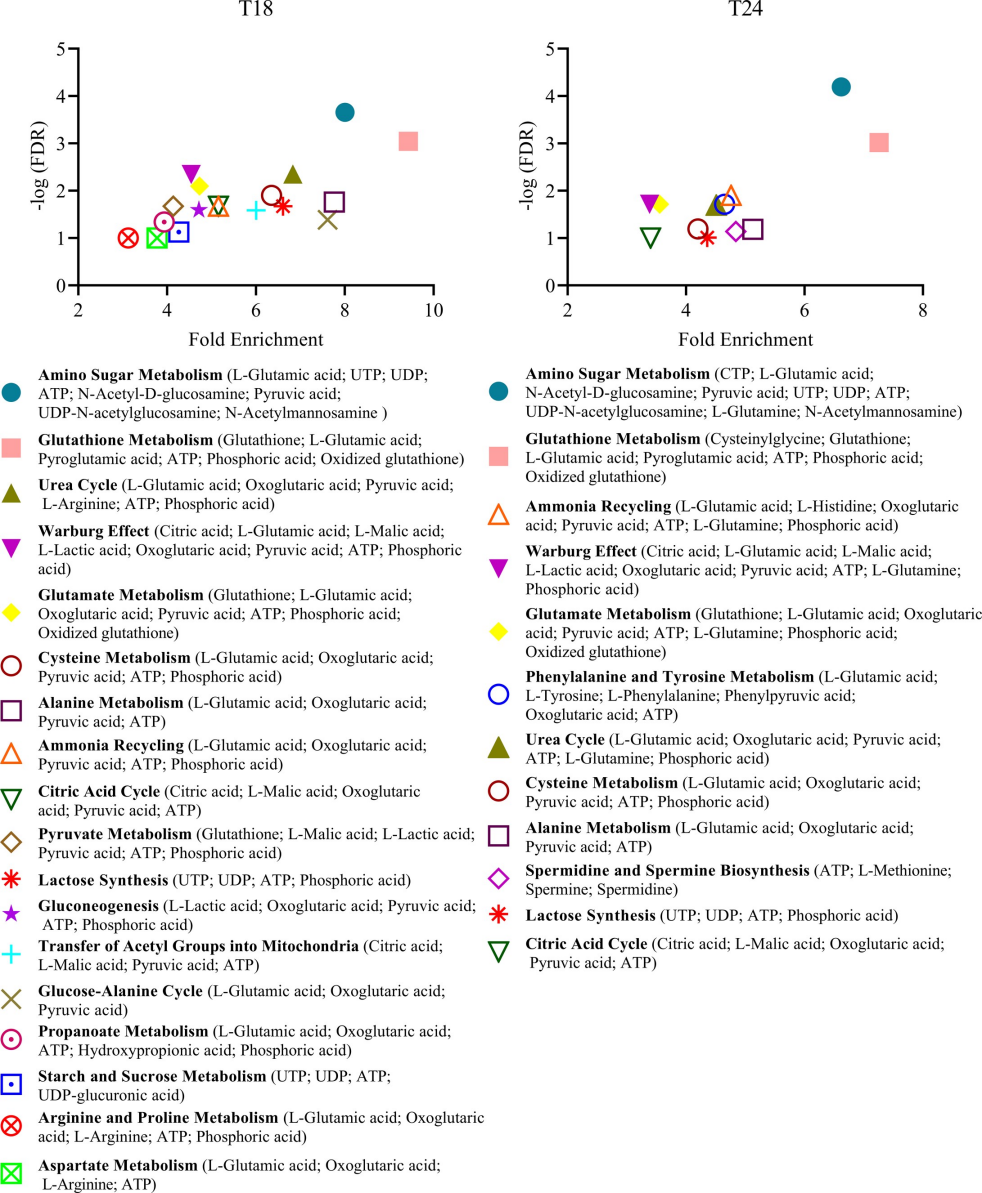
Differentially enriched metabolic sets in HRSV-infected cells compared to the corresponding mock-infected control cells. The list of metabolites identified as differentially expressed in infected cells versus mock-infected cells was used for enrichment analysis using the MetaboAnalyst online tool and the pathway-associated metabolite sets (SMPDB) library. Metabolite sets with a false discovery rate (FDR) ≤ 0.1 are shown.

Our results support the idea that HRSV stimulates the central carbon metabolism since several metabolites from the glycolysis and the TCA cycle were increased after 12–18 hpi, including pyruvic, citric, malic, and oxoglutaric acids (**Figs 2 & 4, S1A File**). Increased levels of glutamine and glutamic acid, precursors of the TCA cycle intermediate α-ketoglutaric acid, were also observed at later times post-infection (**Figs 2 & 4, S1A File**). These findings are supported by the fact that it has been recently reported that urine from infants with HRSV acute respiratory infection was enriched in metabolites from the TCA cycle [24]. Furthermore, infection of A549 epithelial cells with HRSV induces significant changes in components of the electron transport chain complexes and channels, as revealed by quantitative proteomic analysis, confirming the critical role of mitochondrial oxidative phosphorylation in HRSV infection [16, 18, 46].

ATP production is generally supported by the oxidation of glucose through glycolysis, the TCA cycle, and oxidative phosphorylation. However, in highly anabolic states, such as in viral infections that require abundant production of amino acids and nucleotides to build viral proteins and nucleic acids, glutamine may serve as the primary extracellular carbon source to feed the TCA cycle. In this case, glucose is predominantly oxidized through what is called aerobic glycolysis or the Warburg effect [47]. In this process, glucose is converted to pyruvate, but most of this pyruvate is transformed to lactate instead of being oxidized in the mitochondria. This metabolic shift favors the accumulation of glycolysis intermediates for the synthesis of nucleotides, amino acids, and fatty acids necessary for the generation of the viral progeny (**Fig 3**). We have found that levels of lactic acid were increased after 12 hpi (**Figs 2 & 4, S1A File**), indicative of a Warburg effect. Growing evidence support the idea that many DNA viruses (human papillomavirus, HCMV, EBV, KSHV, human adenovirus), but also single-stranded RNA viruses (poliovirus, dengue virus, hepatitis C virus, influenza virus) exploit aerobic glycolysis [1, 3, 48, 49].

### Nucleotide and amino acid levels are increased at times later than 12h post-infection

High amounts of nucleotides and amino acids are necessary for the production of viral nucleic acids and proteins during viral infections. Our results showed increased levels of nucleotides and related compounds at later times of HRSV infection, including ATP, UTP, UDP, GTP, CTP, CMP, and adenosine phosphosulfate (**Figs 2 & 4, S1A File**).

As with nucleotides, the amounts of several amino acids were increased mostly at 24 hpi, such as histidine, phenylalanine, glutamine, methionine, lysine, leucine, tryptophan, tyrosine and glutamic acid (**Figs 2 & 4**).

The increased levels of glutamine, lactate, amino acids, and nucleotides observed in our study strongly suggest that the Warburg effect is taking place during HRSV infection. Therefore, it seems that HRSV, on the one hand, relies on the Warburg effect to promote the synthesis of the building blocks (nucleotides, amino acids, and lipids) for the virus structural components, and, on the other hand, on an anaplerotic reaction in which glutamine substitutes glucose as the predominant extracellular carbon source to feed the TCA cycle and generate energy. Rhinovirus infection also induces enhanced glucose uptake and increased levels of multiple nucleotides, indicating that this virus establishes an anabolic state in infected cells, in some aspects similar to HRSV [50]. Likewise, herpes simplex virus-1 (HSV-1) relies on glutamine uptake to feed the TCA cycle, while glucose is used to drive nucleotide synthesis [51].

In addition to virus production, it is worth noting that HRSV induces a potent cellular antiviral immune response [15, 17, 52]. The concurrence of both the activation of host-cell defenses and the high production of structural components for virion assembly results in a highly anabolic cellular state. Therefore, the increased levels of amino acids and nucleotides (particularly UTP) observed here might be a result of the need for HRSV nucleic acid and protein synthesis, but also for the upregulation of the cellular mRNAs and proteins related to the cell response to virus infection. Type I and II interferons (IFNs) are key components of the immune response against viral infections. It is becoming evident that cellular metabolism and the IFN response are closely interrelated. For example, it has been reported that IFNs increase glucose uptake, glycolysis, aerobic glycolysis, oxidative phosphorylation and ATP production, lipolysis, and reactive oxygen species [53].

### Metabolites related to post-translational modification of proteins are upregulated at later times post-infection

HRSV not only needs nucleotides, amino acids, and lipids to generate viral progeny. To be functional, some HRSV proteins, for example the surface glycoproteins, require post-translational modifications. For example, the fusion (F) and attachment (G) glycoproteins are extensively glycosylated [54]. This process involves the incorporation of distinct amino sugars to the polypeptide backbone mediated by UDP-amino sugar donors. We found that various intermediates of this process, such as pyrimidines, amino sugars, and UDP-amino sugars, were upregulated at later times after HRSV infection, including N-Acetyl-D-glucosamine, UDP-N-Acetylglucosamine, N-Acetylgalactosamine, and N-Acetylmannosamine (**Figs 2 & 4**). Also, the levels of other compounds of amino sugar and nucleotide sugar metabolism were increased at the same time, such as UDP-glucuronic acid and UDP-D-galacturonate (**Figs 2 & 4**). Similarly to HRSV, it has been reported that human cytomegalovirus (HCMV) increases the biosynthesis of different UDP sugars by directing enhanced pyrimidine biosynthesis towards UDP sugar biosynthesis [55].

Also, it has been shown that the HRSV F protein is palmitoylated at cysteine residue 550 [56]. Interestingly, palmitic acid is one of the few lipids that are upregulated at 24 hpi in our study (**Fig 2**). Via the upregulation of UDP sugars and palmitic acid, the production of high amounts of fully matured and functional viral envelope glycoproteins is possible. The relevance of palmitic acid in virus infections was revealed by experiments in which the inhibition of fatty acid synthase (FASN), an enzyme that catalyzes the production of palmitic acid from cytosolic acetyl-CoA and malonyl-CoA, resulted in decreases in the replication of rhinovirus, HRSV, and human parainfluenza virus 3 [57]. The addition of exogenous palmitic acid rescued viral replication, confirming the essential role of this fatty acid in viral infections [57]. These results also illustrate how metabolic pathways could potentially be targeted to treat these infections.

### Lipid metabolism

**Fig 5:**
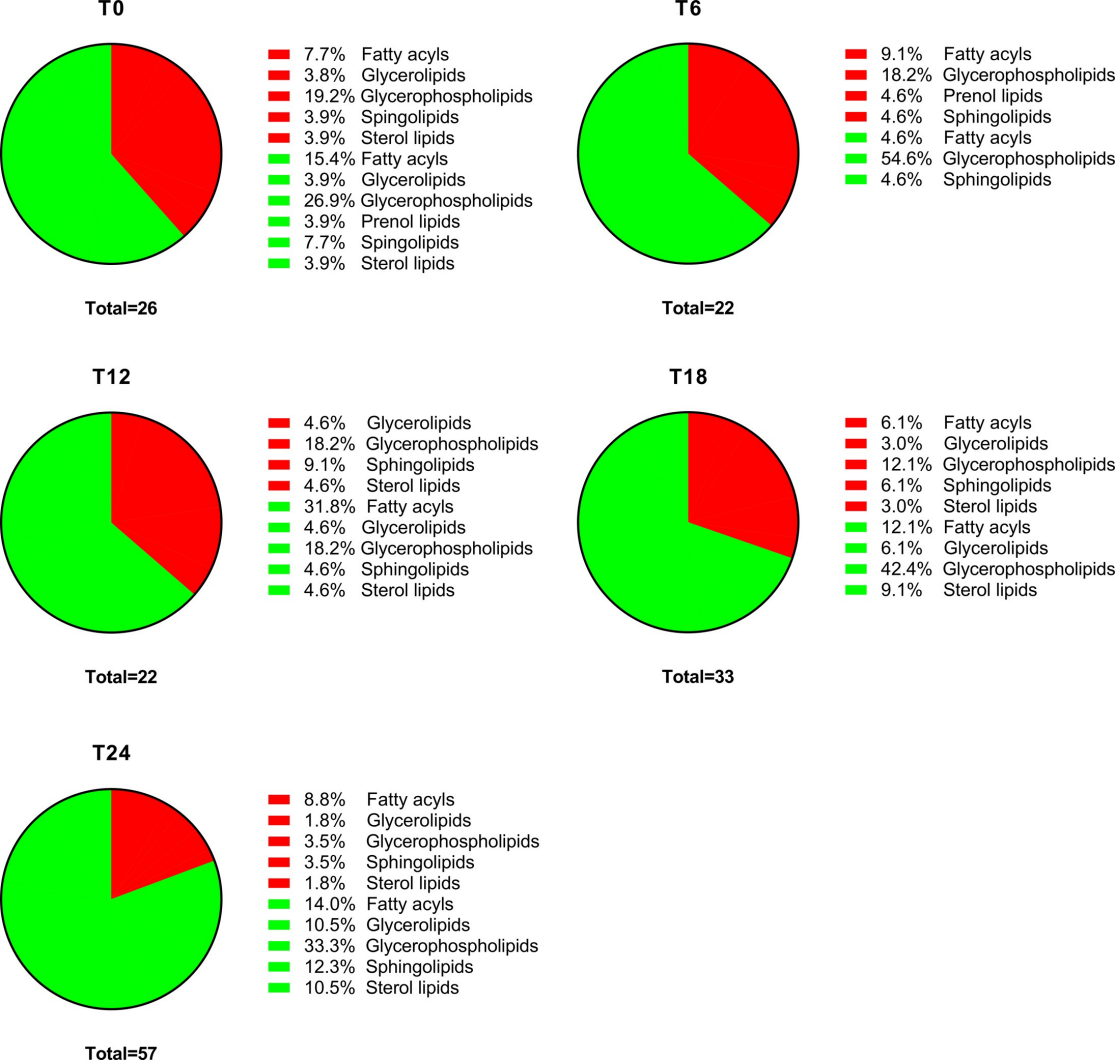
Percentage of differentially expressed lipids in infected versus mock-infected cells. The proportions of upregulated (red) and downregulated (green) lipid categories in infected versus mock-infected cells at each time point post-infection are represented. The total numbers of lipids differentially expressed at each time point are also shown.

HRSV is an enveloped virus, so we expected it to have a profound impact on cellular lipid metabolism. Indeed, the number of different lipids deregulated (up or down) in infected cells increased from 26 at 0 hpi to 57 at 24 hpi (**Fig 5**). Surprisingly, a general downregulation of lipids was observed in infected cells, primarily after 18 hpi (**Fig 5**). Upregulated glycerophospholipids represented 19.2% of total deregulated lipids at 0 hpi, but only 3.5% at 24 hpi (**Fig 5**). In contrast, the percentage of upregulated fatty acyls and sphingolipids remained approximately constant throughout the infection (**Fig 5**).

Additionally, we observed a decrease in palmitoylcarnitine and stearoylcarnitine levels at 24 hpi (**Fig 2**). Since carnitine assists in the transfer of long-chain fatty acids from the cytoplasm to mitochondria for subsequent β-oxidation [58], this result suggests that β-oxidation was reduced in infected cells. However, as discussed below, other mechanisms may promote the transfer of fatty acids to mitochondria.

Since most enveloped viruses induce lipogenesis [2], the generalized downregulation of lipid levels in HRSV-infected cells at later times post-infection was unexpected. A possible explanation is that HRSV infection leads to increased lipid degradation. Some viruses induce a specific autophagy called lipophagy that processes lipids from lipid droplets to release free fatty acids for β-oxidation and energy generation [59, 60]. HRSV induces autophagy to increase its replication through activation of the AMP-activated protein kinase/mammalian target of rapamycin (AMPK-mTOR) pathway [61]. Although lipophagy has not been explicitly studied in HRSV infections, the AMPK-mTOR pathway has been shown to be activated by the dengue virus to stimulate proviral lipophagy [60], suggesting that the same process may take place in HRSV infections. Besides lipophagy, lipid droplets can be broken down by other mechanisms, such as triglyceride lipase-mediated lipolysis. In addition to triglycerides and cholesteryl esters, diverse glycerophospholipids and glycosphingolipids are also hydrolyzed in the lysosomes by phospholipases and acid hydrolases [62].

### Oxidative stress: Glutathione system and polyamines

Several reports have shown that HRSV induces oxidative stress in infected cells, resulting in increased levels of oxidized glutathione [25–28]. Actually, increased production of mitochondrial reactive oxygen species (ROS) favors HRSV production, suggesting new potential therapeutic approaches against this virus [63]. The cells usually upregulate the synthesis of glutathione and polyamines (putrescine, spermidine, and spermine) to counteract that stress. In our study, an increase in glutathione and oxidized glutathione, indicative of oxidative stress, was observed at 18 and 24 hpi (**Figs 2 & 4**). Likewise, spermidine and spermine levels increased at 24 hpi (**Figs 2 & 4**). In addition to protecting cells from oxidative stress, it is worth noting that polyamines support the replication of several RNA viruses by binding viral RNA and accelerating the translation of viral proteins [64].

Lipid droplets accumulate in cells under oxidative stress, and oxidation of fatty acids from lipid droplets not only provides energy but also protects cells from the stress by maintaining the glutathione-dependent antioxidant system [65]. Therefore, our results suggest that HRSV may promote lipid degradation to obtain additional energy for virus production but also to control excessive oxidative stress.

## Conclusion

In conclusion, our data show how HRSV infection affects cell metabolism to ensure an adequate supply of energy and structural components for virus replication. Cells, however, respond to the infection with countermeasures aimed to restrict virus growth. Manipulation of the metabolism of infected cells may be a potential approach for developing effective treatments against this human-relevant pathogen, and several inhibitors of metabolic processes are already in use.

## Supporting information

S1 Fig Levels of metabolites in cellular extracts. Metabolites presenting statistically significant differences between infected and mock-infected cells were selected, and a heatmap was created with the identified metabolites. The heatmap shows the individual values from five independent experiments for each time point (0, 6, 12, 18 and 24 hpi). Levels correspond to the compound area and are represented as log_2_(infected/uninfected).
(PDF)

S1A File Top 25 enrichment categories upregulated in infected cells versus non-infected cells. The list of metabolites identified as differentially expressed in infected cells versus mock-infected cells was used for enrichment analysis using MetaboAnalyst and the pathway-associated metabolite sets (SMPDB) library.
(XLSX)

S1B File Top 25 enrichment categories downregulated in infected cells versus non-infected cells. The list of metabolites identified as differentially expressed in infected cells versus mock-infected cells was used for enrichment analysis using MetaboAnalyst and the pathway-associated metabolite sets (SMPDB) library.
(XLSX)
