# Supplementary figures and images for: Metabolic changes during respiratory syncytial virus infection of epithelial cells
Source: PLoS One. 2020 Mar 26;15(3):e0230844. doi: 10.1371/journal.pone.0230844 (PMC7098640; doi:10.1371/journal.pone.0230844)

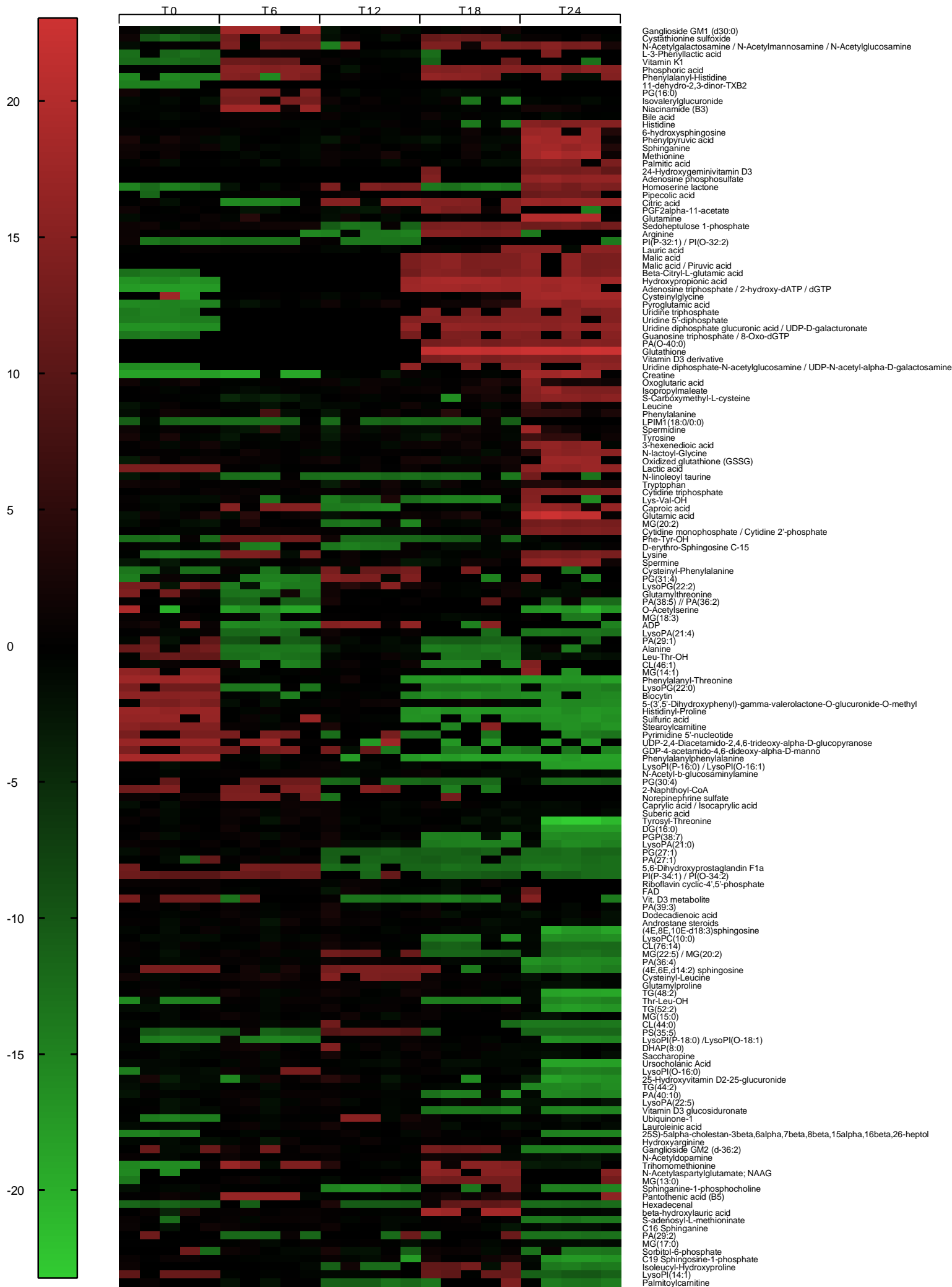

Supplement: S1 Fig — Metabolites presenting statistically significant differences between infected and mock-infected cells were selected, and a heatmap was created with the identified metabolites. The heatmap shows the individual values from five independent experiments for each time point (0, 6, 12, 18 and 24 hpi). Levels correspond to the compound area and are represented as log2(infected/uninfected). (PDF) [file pone.0230844.s001.pdf]
